# Sensitivity analysis of unsafe behaviors in the spinning and weaving factories: Exploring the association with burnout and resilience using Bayesian networks

**DOI:** 10.1371/journal.pone.0326883

**Published:** 2025-07-07

**Authors:** Roozbeh Azimi, Saleh Al Sulaie, Saeid Yazdanirad, Amir Hossein Khoshakhlagh, Rosanna Cousins, Fatemeh Kazemian

**Affiliations:** 1 Department of Occupational Health, School of Health, Tehran University of Medical Sciences, Tehran, Iran; 2 Department of Mechanical and Industrial Engineering, College of Engineering and Computing in Al-Qunfudah, Umm Al-Qura University, Makkah, Saudi Arabia; 3 Social Determinants of Health Research Center, Shahrekord University of Medical Sciences, Shahrekord, Iran; 4 Social Determinants of Health (SDH) Research Center, Kashan University of Medical Sciences, Kashan, Iran; 5 Department of Psychology, Faculty of Human and Digital Sciences, Liverpool Hope University, Liverpool, United Kingdom; 6 Student Research Committee, Kashan University of Medical Sciences, Kashan, Iran; Shahrood University of Medical Sciences, ISLAMIC REPUBLIC OF IRAN

## Abstract

Job burnout and resilience skills are factors that can affect safety performance in the workplace. However, the contribution of these variables to unsafe behaviors through various paths has not been determined. This study aimed to investigate the association of three burnout dimensions and resilience with safety compliance and safety performance using Bayesian network modeling. This research was performed with cross-sectional design. Participants were 200 employees working in some spinning and weaving factories. Participants provided responses to printed survey items during work rest periods. The survey comprised a demographic information section, validated Persian versions of the Connor–Davidson resilience scale, the Maslach burnout questionnaire, and the safety behavior assessment. The Bayesian network was analyzed using version 2.3 of the GeNIe academic software. At the high state with a probability of 100% for each of the three burnout variables: depersonalization, emotional exhaustion, personal accomplishment, and (poor) resilience, the probability of poor safety compliance increased by 16%, 16%, 7%, and 24% and the probability of poor safety participation rose by 6%, 12%, 29%, and 17%, respectively. All variables with a probability of 100% also elevated the likelihood of diminished safety compliance and reduced safety participation by 51% and 34%, respectively. Each of the three dimensions of burnout can be associated with changes in resilience, safety compliance, and safety participation. Resilience plays a significant role in mediating the association between burnout dimensions and unsafe behaviors.

## Background

One of the factors that threatens employees’ health, productivity, and organizational performance is occupational accidents [1]. Early attempts to understand the context of occupational accidents focused on identifying the so-called ‘accident-prone personality’, however when it was found that there were very few accident-repeaters the focus shifted elsewhere [2]. Heinrich (1931) stated that the cause of 88% of accidents in the workplaces is unsafe actions, and cause of 10% of accidents is unsafe conditions (The remaining 2% were accounted for in terms of ‘acts of God’.) [3,4].

Certainly, important historical disasters such as Three Mile Island, Chernobyl, Bhopal, and Flensburg confirmed the significant involvement of human factors in industrial accidents. Also, unsafe behaviors can escalate the consequences of an unsafe condition. A pertinent example of this is found in reports of the fires at the Texaco Refinery, Milford Haven in 1994 [5]. Although the initial fire was caused by a lightning strike, the subsequent explosion and multiple secondary fires were a result of complacency and a history of unsafe behaviors among employees of all ranks. The cost of the damage, and legal proceedings (brought by the UK Health & Safety Executive due to many ‘duties of care’ issues), led to fines and costs of more than £343,000 (in 1996). Despite being a high-hazard organization, Texaco Refinery had no human factors expertise on site, and a significant number of recommendations followed from the United Kingdom Health & Safety Executive which amounted to a thorough review of work practices and work rosters which indicated burnout and fatigue problems from working seven consecutive nights [6].

Predicting and controlling human behavior in hazardous conditions remains an important factor in preventing occupational accidents [7]. Many studies have been carried out to discover the causes and control the recurrence and prevention of accidents, but there are still many outstanding questions in understanding underlying predictors of behavior that have not been fully explored in terms of their associations with accidents and unsafe behaviors. One potentially important predictor is burnout syndrome. Burnout is described as an occupational phenomenon that is caused by chronic work-related stress that has not been managed appropriately [8]. This condition is characterized by an overwhelming sense of exhaustion, a disconnection from one’s profession, and a diminished effectiveness at work [9,10]. Burnout syndrome is usually diagnosed using the Maslach Burnout Inventory (MBI). Research in various industries on both white-collar and blue-collar workers has implicated burnout as a consequence of inadequately managed chronic workplace stress [11–14]. Burnout is associated with increased long-term physical and psychological risks, increased sick leave, absenteeism, job turnover, and poor efficiency on the job [15]. Altogether, the consequences of burnout are considered one of the most critical issues in today’s complex industrial world [16,17]. Burnout can influence mental health, decrease focus, and thereby increases unsafe behaviors among employees [18].

Resilience can regulate association between burnout and unsafe behaviors. Resilience has defined resilience as a dynamic process, associated with reactive recovery from challenging situations, and marked by individual protective factors that promote good outcomes [19–21]. Whilst not referring directly to workplace behaviors, Youseff & Luthans (2007), provided evidence that resilience is related to workplace performance. This is important for managing the potential for unsafe behaviors among employees in the workplace. Particularly, considering that resilience skills can be developed, and as such resilience is amenable to proactive learning and positive progress through training interventions [19,22,23].

There is some prior research that has considered associations between burnout variables, resilience, and unsafe behaviors. For example, Nahrgang, Morgeson & Hofmann (2011) performed a meta-analytic investigation on the relationships between burnout and safety outcomes and observed that burnout was negatively related to working safely [24]. Similarly, Vévoda et al. (2016) found substantial negative associations between burnout dimensions and psychological safety at work [25], and Shojafard, Pour Sedegh, Shahr Ashroub & Zangisheh (2014) investigated the association between burnout and resilience among medicine personnel and found that there is a substantial association between these variables [26]. Shakerinia & Mohammadpour (2010) found that resiliency is a strong variable to decrease stress and burnout in nursing [27], and Wehbe, al Hattab & Hamzeh (2016) showed that higher resilience has a link with prevalent risks and better actual safety performance [28]. Li et al. (2022) investigated the impact of resilience on safety behavior and observed that resilience positively influences safety attitude and safe behavior [29]. However, to our knowledge, the interactive associations of these variables in a model have not been previously examined. There are analysis techniques for examining this association using Bayesian networks. A Bayesian network is a probabilistic graphical model that depicts variables of interest in terms of their conditional dependencies [30]. They possess a particular skill in assessing an event to ascertain the likelihood of it being influenced by multiple possible factors [31]. Bayesian networks have been used to analyze relations between variables in numerous fields such as safety, health, and decision support systems, and they were deemed suitable for use in the presented study.

The aim of the study was, for the first time, to examine the associations between job burnout dimensions and unsafe behavior with the mediator role of resilience. The study took place in the spinning and weaving industries in Kashan city, Iran. These industries are now highly demanding technical industries and a setting in which many accidents happen each year.

Previous research (e.g., Mohammadinejad et al., 2019 [32] and Nag, Nag & Vyas, 2009 [33]) has confirmed that non-observance of safety principles is one of the causal factors, and unsafe behaviors have caused many workers to become permanently disabled, which in turn leads to other negative consequences associated with economic and psychosocial damage.

## Methods

This study was performed with a cross-sectional design. The study received ethical approval from the research ethics committee of Kashan University of Medical Sciences (IR.KAUMS.MEDNT.REC.1402.155). The start and end dates were 29 January 2024 and 20 November 2024, respectively.

### Participants

Participants were recruited from several spinning and weaving industries. The inclusion criteria were aged 18–60 years, a minimum of one year of employment in their factory, and an absence of psychiatric disorders. The exclusion criterion was an incomplete survey submission. Invitations were sent out to employees with information about the study using random sampling until a total of 200 employees who met the inclusion criteria. They completed the survey used for data collection. In practice, 240 eligible employees were invited to join the study, and thus participation rate was equal to 83.3 percent. Participants provided written consent during the enrollment process.

### Data collection

To gather data related to study goals, the participants were asked to complete the printed survey instrument comprised of demographic information, the Connor–Davidson Resilience Scale, the Maslach Burnout questionnaire, and the Safety Behaviors Assessment. This was completed during a rest period.

### Demographical information questionnaire

The demographic information questionnaire included several questions on age, work experience, gender, and education level.

### Job burnout questionnaire

This questionnaire was developed by Maslach & Jackson (1984) and is used to measure occupational burnout. This questionnaire contains 22 items and three subscales: emotional exhaustion (EE; 9 items), depersonalization (DP; 5 items), and personal accomplishment in the framework of professional activity (PA; 8 items). Each item was evaluated using a 7-point Likert scale ranging from 0 to 6, with subscale scores subsequently computed. For emotional exhaustion (EE) and depersonalization (DP), higher scores indicate greater levels of burnout, whereas for personal accomplishment (PA), lower scores suggest increased burnout. Maslach documented internal consistency coefficients of 0.9 for EE, 0.79 for DP, and 0.71 for PA [34]. The Persian version of this questionnaire demonstrated robust psychometric qualities, as verified by Moalemi et al. in 2018, who found Cronbach’s alpha values of 0.85 for EE, 0.71 for DP, and 0.76 for PA [35]. Therefore, the validity and reliability of the Persian version of this questionnaire similar to original version were confirmed by Moslemi et al. [35].

### The Connor–Davidson resilience scale

This questionnaire was developed by Connor & Davidson (2003) to evaluate individual resilience. The instrument comprises 25 questions distributed across five distinct dimensions: personal competence (8 questions), tolerance of negative affect and stress enhancement (5 questions), positive acceptance of change (7 questions), sense of control (3 questions), and spiritual influence (2 questions) [36]. Responses to all items were provided using a five-point Likert scale, which varied from 0 (not true at all) to 4 (almost always true). The sum of item scores related to each dimension provides a dimension score, and the sum of the dimension scores provides a measure of resilience. A higher score shows greater resilience. Connor & Davidson (2003) reported a reliability coefficient of 0.87 [36,37]. Derakhshanrad et al. confirmed that validity and reliability of the Persian version of this questionnaire [38]. The reliability of the Persian translation of this scale has been reported as 0.892 confirming its suitability for this study [38].

### Safety behavior assessment

Mahdinia et al. (2016) developed and confirmed the reliability of a questionnaire (presented in Persian) aimed at assessing safety behaviors within Iran. This instrument consists of 23 items divided into two categories: safety compliance, which includes 12 items, and safety participation, encompassing 11 items. Responses to each item are measured on a five-point scale of agreement, where 1 indicates ‘never’ and 5 signifies ‘always’. The sum of the scores of the questions related to each dimension shows the total score of that dimension. Higher scores are related to better safety behavior. Mahdinia et al. confirmed the validity and reliability of this questionnaire and reported a Cronbach’s alpha coefficient of 0.902 [39].

### Statistical analyses

Statistical analyses were performed using version 24 of the SPSS software. The alpha Cronbach’s alpha coefficient of the questionnaires were also computed. Descriptive statistics were calculated, and participants were classified into either a ‘low’ or ‘high’ category based on the median values of the six variables examined in this research (EE, DP, PA, resilience, safety compliance, and safety participation), with ‘low’ being the desired status for all variables. That is, of the burnout variables: for EE low indicated below the median EE score; for DP low indicated below the median DP score, for PA low indicated reduced PA, and a score above the median. For resilience low indicated reduced resilience and a score above the median and similarly, for safety compliance and safety participation, high indicated a score above the median and poor safety compliance and poor safety participation.

The method of expectation-maximization was utilized to address the issue of missing data. The analysis of the Bayesian network was conducted using GeNIe academic software, version 2.3. Within the software’s model, connections were established among six key variables identified in this research: emotional exhaustion (EE), depersonalization (DP), personal accomplishment (PA), resilience, safety compliance, and safety participation. The expectation-maximization algorithm was specifically employed for the estimation of parameters within the Bayesian network, providing a deterministic approach that is effective in approximating unknown parameters, particularly useful in cases with incomplete or insufficient data [40].

First, a theoretical model was drawn based on the relationships assumed in the GeNIe academic software. In this model, the data needs to be entered into the model qualitatively based on the classification performed at different levels (low and high). For this purpose, the probability of each of the states was obtained by crosstabs analysis in SPSS software based on the conditional probability table, and then these values were entered into the tables considered in the software for the GeNIe model. So, upon constructing the theoretical framework of the Bayesian network, a conditional probability table was generated through the model using the expectation-maximization algorithm [41]. For the sensitivity analysis of the model, the intended variables were placed on the worst state or the best state with a probability of 100 percent to determine how the other variables of the model change. This is called univariate sensitivity analysis. The largest change in the conditional probability of each variable indicates the highest association with the variable with a probability of 100 percent. In addition, an increase or decrease in the probabilities indicates a positive or negative relationship between the variables. This sensitivity analysis can also be performed for several variables with a probability of 100 percent, which is called multivariate sensitivity analysis. A Delta p sensitivity analysis was conducted to assess the association between these variables [42]. Categories analyzed included both low and high levels of EE, DP, PA, resilience, safety compliance, and safety participation. The sensitivity analysis extended to all possible individual states and combinations of these variables. To validate the model’s accuracy, a 10-fold cross-validation analysis was performed. The dataset was divided randomly into ten subsets; nine of these subsets were used for training the Bayesian network model, and the tenth subset served for validation purposes [43].

## Results

The demographic characteristics of 200 spinning and weaving employees who participated in this study are reported in Table 1. The average age of the participants was 35.6 years with a standard deviation of 11.71, and their average work experience was 10.05 years with a standard deviation of 5.87. Table 2 displays the frequencies of the variables under study, while Table 3 illustrates the conditional probabilities associated with unsafe behaviors.

**Table 1:** Demographic characteristic of the subjects.

Parameter		Frequency	Relative frequency
**Age (years)**	Less than 30 years	75	35.7
	30 to 50 years	118	56.2
	More than 50 years	17	8.1
**Career length (years)**	Less than 5 years	53	25.2
	5 to 15 years	141	67.1
	More than 20 years	16	7.7
**Sex**	Men	197	93.8
	Women	13	6.2
**Education level**	Under bachelor’s degree	188	89.5
	Bachelor’s degree	20	9.5
	Above Bachelor’s degree	2	1.0

**Table 2:** Frequency of the studied variables. Low is the desired state for all variables.

Parameter		Frequency	Relative frequency
**Emotional exhaustion (EE)**	Low	108	54
	High	92	46
**Depersonalization (DP)**	Low	94	47
	High	106	53
**Reduced Personal Accomplishment (PA)**	Low	121	60.5
	High	79	39.5
**Poor Resilience**	Low	136	68
	High	64	32
**Poor Safety compliance**	Low	109	54.5
	High	91	45.5
**Poor Safety participation**	Low	88	44
	High	112	56

**Table 3:** Conditional probability table for unsafe behaviors. Low is the desired state for all variables.

EE	DP	Reduced PA	Poor Resilience	Poor Safety Compliance	Poor Safety Participation	
					Low	High
**Low**	Low	Low	Low	Low	1.00	0.00
				High	1.00	0.00
			High	Low	0.481	0.519
				High	0.077	0.923
		High	Low	Low	0.800	0.200
				High	0.286	0.714
			High	Low	0.00	1.00
				High	0.00	1.00
	High	Low	Low	Low	0.75	0.25
				High	0.00	1.00
			High	Low	0.50	0.50
				High	0.50	0.50
		High	Low	Low	0.625	0.375
				High	0.333	0.667
			High	Low	0.50	0.50
				High	0.50	0.50
**High**	Low	Low	Low	Low	0.50	0.50
				High	0.444	0.556
			High	Low	0.50	0.50
				High	0.50	0.50
		High	Low	Low	0.60	0.40
				High	0.167	0.833
			High	Low	0.50	0.50
				High	0.50	0.50
	High	Low	Low	Low	0.50	0.50
				High	0.50	0.50
			High	Low	0.00	1.00
				High	0.00	1.00
		High	Low	Low	0.571	0.429
				High	0.429	0.571
			High	Low	0.00	1.00
				High	0.048	0.952

The alpha Cronbach coefficients related to job burnout questionnaire, the Connor–Davidson resilience scale, and safety behavior assessment questionnaire were computed by 0.805, 0.963, and 0.878. These results show that the reliability of the used tools were confirmed in the samples studied.

**Fig 1:**
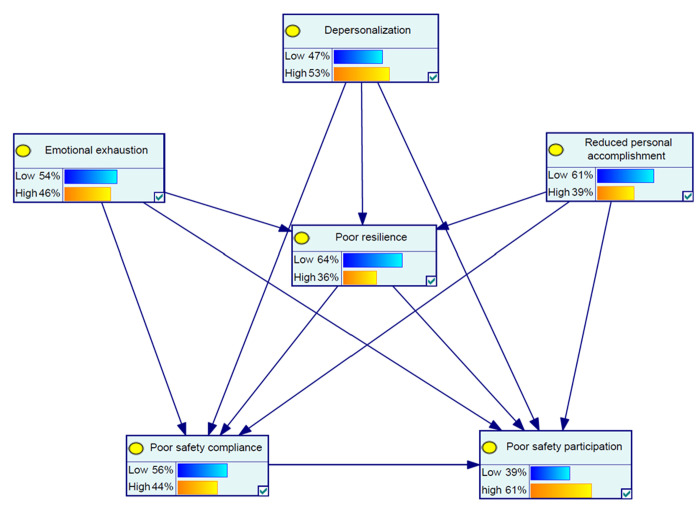
The conceptual framework for the marginal probabilities of variables as per the Bayesian network model.

Fig 1 shows the theoretical model for the marginal probabilities of the variables according to the Bayesian network model. Table 4 provides the results of the univariate sensitivity analyses. Overall, at the high state with a probability of 100% for burnout variables EE, DP, and PA, the probability of poor resilience positively changed by 12%, 6%, and 10%, respectively. Moreover, at the high state with a probability of 100% for each of the variables of EE, DP, PA, and poor resilience, the probability of poor safety compliance increased by 16%, 16%, 7%, and 24%, and the probability of poor safety participation was raised by 6%, 12%, 29%, and 17%, respectively. Poor safety compliance with a probability of 100% also could increase the probability of high EE, DP, and PA, poor resilience, and poor safety participation by 17%, 19%, 7%, 19%, and 21%, respectively. Poor safety participation with a probability of 100% also could increase the probability of EE, DP, PA, poor resilience, and poor safety compliance by 5%, 10%, 19%, 10%, and 1%, respectively.

**Table 4:** The findings of univariate sensitivity analyses. Low is the desired state for all variables.

Parameter	Level	Low (100%)						High (100%)					
		Emotional Exhaustion	Depersonalization	Reduced Personal Accomplishment	Poor resilience	Poor safety compliance	Poor safety participation	Emotional exhaustion	Depersonalization	Reduced Personal Accomplishment	Poor resilience	Poor safety compliance	Poor safety participation
**Emotional Exhaustion**	Low	–	0	0	+9	+13	+7	–	0	0	−17	−17	−5
	High	–	0	0	−9	−13	−7	–	0	0	+17	+17	+5
**Depersonalization**	Low	0	–	0	+5	+15	+16	0	–	0	−9	−19	−10
	High	0	–	0	−5	−15	−16	0	–	0	+9	+19	+10
**Reduced Personal Accomplishment**	Low	0	0	–	+6	+5	+29	0	0	–	−12	−7	−19
	High	0	0	–	−6	−5	−29	0	0	–	+12	+7	+19
**Poor Resilience**	Low	+11	+7	+7	–	+15	+16	−12	−6	−10	–	−19	−10
	High	−11	−7	−7	–	−15	−16	+12	+6	+10	–	+19	+10
**Poor Safety Compliance**	Low	+14	+18	+5	+13	–	+24	−16	−16	−7	−24	–	−1
	High	−14	−18	−5	−13	–	−24	+16	+16	+7	+24	–	+1
**Poor Safety Participation**	Low	+5	+14	+19	+10	+17	–	−6	−12	−29	−17	−21	–
	High	−5	−14	−19	−10	−17	–	+6	+12	+29	+17	+21	–

Table 5 presents the findings from multivariate sensitivity analyses. The analysis reveals that when considering two variables at a certainty of 100%, the greatest increase in the likelihood of significantly low safety compliance (39%) and markedly low safety participation (37%) can be attributed to high emotional exhaustion associated with poor resilience, and high reduced personal accomplishment associated with poor resilience, respectively. In scenarios involving three variables at a probability of 100%, the most substantial increases in the likelihood of high poor safety compliance (51%) and poor safety participation (34%) were observed with high emotional exhaustion, high depersonalization, and high reduced personal accomplishment. In all cases where variables were certain at 100%, there was an increase in the likelihood of very low safety compliance and participation by 51% and 34%, respectively.

**Table 5:** The findings of multivariate sensitivity analysis.

Distribution (100%)				Poor Safety Compliance (%)		Poor Safety Participation (%)	
EE (low)	DP (low)	Reduced PA (low)	Resilience (low)	Low	High	Low	High
✔	✔			+26	−26	+25	−25
✔		✔		+10	−10	+23	−23
✔			✔	+17	−17	+6	−6
	✔	✔		+33	−33	+36	−36
	✔		✔	+27	−27	+28	−28
		✔	✔	+10	−10	+26	−26
✔	✔	✔		+35	−35	+44	−44
✔	✔		✔	+26	−26	+29	−29
	✔	✔	✔	+34	−34	+41	−41
✔	✔	✔	✔	+40	−40	+61	−61
EE (high)	DP (high)	Reduced PA (high)	Resilience (high)	Low	High	Low	High
✔	✔			−38	+38	−11	+11
✔		✔		−38	+38	−37	+37
✔			✔	−39	+39	−29	+29
	✔	✔		−10	+10	−37	+37
	✔		✔	−35	+35	−15	+15
		✔	✔	−38	+38	−37	+37
✔	✔	✔		−51	+51	−34	+34
✔	✔		✔	−50	+50	−22	+22
	✔	✔	✔	−51	+51	−34	+34
✔	✔	✔	✔	−51	+51	−34	+34

Table 6 presents the impact values associated with the interrelationships among the variables in the model. Regarding resilience, the highest influence values were found in EE and reduced PA. Regarding poor safety compliance, the highest influence values were seen in DP and EE. Regarding poor safety participation, the highest influence value was derived from low reduced PA.

**Table 6:** The impact values associated with the interrelationships among the variables in the model.

Parent	Child	Average	Maximum	Weighted
**Reduced PA**	Poor resilience	0.631	1	0.631
**Reduced PA**	Poor safety compliance	0.202	0.401	0.202
**Reduced PA**	Poor safety participation	0.376	0.923	0.376
**Poor safety compliance**	Poor safety participation	0.165	0.75	0.165
**Emotional exhaustion**	Poor resilience	0.631	1	0.631
**Emotional exhaustion**	Poor safety compliance	0.278	0.568	0.278
**Emotional exhaustion**	Poor safety participation	0.326	1	0.326
**Depersonalization**	Poor resilience	0.109	0.28	0.109
**Depersonalization**	Poor safety compliance	0.326	0.6	0.326
**Depersonalization**	Poor safety participation	0.156	0.714	0.156
**Poor resilience**	Poor safety compliance	0.298	0.618	0.298
**Poor resilience**	Poor safety participation	0.354	0.556	0.354

**Fig 2:**
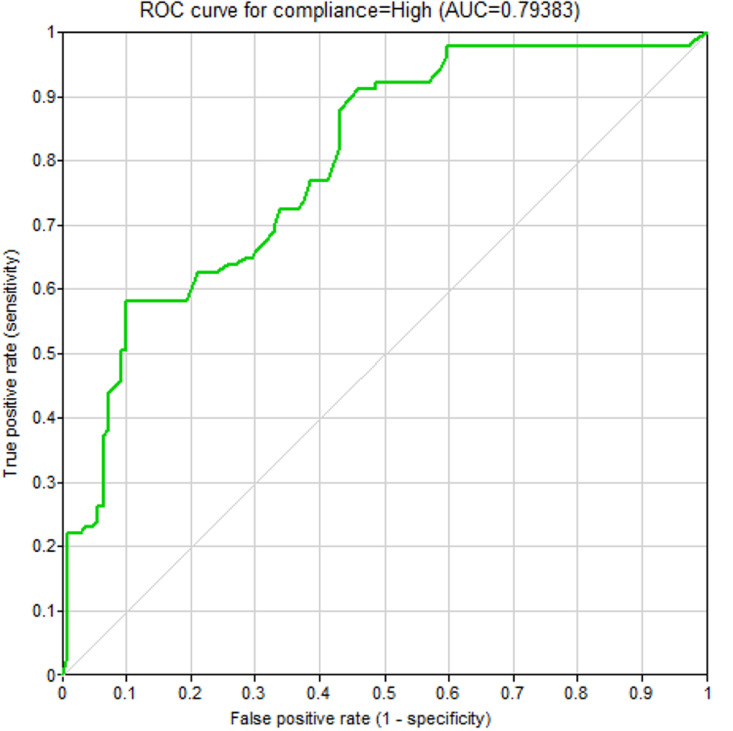
Receiver Operating Characteristics curve demonstrating the validity of the model.

A receiver operating characteristics (ROC) curve was drawn to examine the validity of the fitted Bayesian model (see Fig 2). The area under the ROC curve was equal to 0.794 confirming that the predictive ability of the model was very good.

A ROC curve was constructed to assess the accuracy of the Bayesian model implemented (see Fig 2). The area beneath this curve measured 0.794, indicating a high level of predictive performance for the model. The sensitivity (0.725), specificity (0.661), and accuracy (0.705) values confirm the validity of the model.

## Discussion

The aim of the study was, for the first time, to fully examine the associations between job burnout factors, resilience, safety participation, and safety compliance in the occurrence of unsafe behaviors. All burnout factors can significantly associated with change in the probability of resilience, safety participation, and safety compliance. An understanding of these relationships is fully appreciated when we first consider how these burnout dimensions are represented in the workplace. In their review of burnout measures, Edú-Valsania, Laguía, & Moriano (2022) characterized depersonalization as an emotional state of detachment, a lack of concern, and a sense of indifference toward one’s work and the individuals served by it [44]. This condition manifests through adverse or improper attitudes and conduct, such as irritability, a decline in idealism, and a tendency to avoid interactions with peers and/or clients. Emotional exhaustion is identified by feelings of depletion, weariness, and a diminished capacity due to the mental exertion required by their job. This leads to challenges in adjusting to the workplace because individuals do not possess the necessary emotional reserves to manage their professional responsibilities [44]. Reduced personal accomplishment is evident in a negative assessment of one’s professional capabilities and questioning one’s effectiveness in their role. It is also associated with a pessimistic view of outcomes, reduced productivity and skills, low morale, and weakened abilities to handle work-related stress [44].

The results confirmed the involvement of burnout in unsafe behaviors, and at the high state of emotional exhaustion, depersonalization, and decreased personal accomplishment, the likelihood of poor resilience went up, particularly for emotional exhaustion. This may be because emotional exhaustion is an important factor. Schaufeli & Buunk (2003) argued that emotional exhaustion affects the other two dimensions, and that decreased personal accomplishment is a consequence of the other two dimensions [45]. This suggests that burnout may first be realized by emotional exhaustion and then, depersonalization creeps in, and reduced personal accomplishments are created [45]. Our evidence supports the tenet that emotional exhaustion can more rapidly than the other two burnout dimensions change individual resilience, and as such the signs of emotional exhaustion in employees should also be considered as a risk for unsafe behaviors by dint of its relationships with other relevant variables. The results of related research corroborate the findings of the present study and the associated discussion. For instance, a systematic review and meta-analysis by Deldar et al. (2018) explored the connections between resilience and various aspects of burnout. They found that resilience correlated negatively with emotional exhaustion and depersonalization at −0.62 and −0.41, respectively, and positively with personal accomplishment at 0.25 [46]. Similarly, Ríos-Risquez et al. (2016) examined how resilience related to burnout among nursing students, noting a significant inverse relationship between resilience and emotional exhaustion [47].

The results of the current study illustrate that, at the high state for each of the parameters of emotional exhaustion, depersonalization, reduced personal accomplishment, and poor resilience, the probability of poor safety compliance increased by 16%, 16%, 7%, and 24%, which confirms the importance of emotional exhaustion, depersonalization, and resilience in safety performance in line with previous findings. Baier et al. (2018) explored the link between burnout and safety outcomes among employees, identifying that depersonalization and emotional exhaustion are predictors of safety behavior [48]. Similarly, Salyers et al. (2017) investigated how emotional exhaustion, depersonalization, and personal accomplishment relate to safety within a healthcare environment [49].

An interesting result in the present study, which adds to the establishment of these relationships, is that resilience can strongly mediate the effects of burnout factors on safety compliance. Park and Eo (2016) previously demonstrated that resilience partially mediates the relationship between social capital and safety awareness, a finding that has been corroborated in our research [50]. Chen et al. (2017) explored how individual resilience impacts interpersonal conflicts at work and the subsequent effects on safety outcomes among construction workers. They discovered that higher levels of individual resilience were significantly associated with fewer interpersonal conflicts, which, in turn, led to reduced occurrences of physical safety incidents [20]. These findings align with the results obtained from our current study.

The results of the present study indicated that at the high state for each of the variables of emotional exhaustion, depersonalization, decreased personal accomplishment, and poor resilience, the probability of high poor safety participation was raised. Also, greatest problem in terms of this aspect of safety performance was the influence of burnout from reduced personal accomplishment. The importance of resilience in safety outcomes was mentioned above, however, the role of personal accomplishment in safety has had less attention. Corbeanu et al. (2023) have recently performed a meta-analysis on the association between burnout measures and job performance and concluded that whilst all three were important, the relationship between reduced personal accomplishment and job performance was strongest [51]. Similarly, Zarei et al. (2016) demonstrated that within the various aspects of occupational burnout, the dimension of diminished personal accomplishment, considering both its intensity and occurrence, exhibited the strongest correlation with the elements of safety climate [52]. These findings show the importance of personal accomplishments in voluntary participation in the implementation of safety measures. Moreover, the multivariate sensitivity analysis in the present study showed that it is insufficient to focus on a single burnout measure, as all three in a combination of variables impacted on safety compliance and safety participation.

Whilst we recognize that a limitation of this study is not being able to include other variables likely to be associated with safety outcomes, such as job satisfaction, social support, organizational processes, cultural influences, work environment factors, personality, motivation, coping abilities, personal psychological traits, and job stress, into the model in the present study, nevertheless, the importance of burnout and its potentiation by resilience in understanding unsafe behaviors is endorsed. It is recommended that future studies extend the involvement of occupational variables in unsafe behaviors to further elucidate our understanding and reduce workplace accidents. Moreover, as another limitation, this study was only performed within the spinning and weaving factories in Iran that reduces the generalizability of the results to other industries or different cultural contexts. Therefore, it is supposed that these relations are conducted in other industries of various countries. Furthermore, data were collected at a single point in time, and the observed relationships do not imply causality.

## Conclusion

Totally, all three burnout factors can be associated with resilience, safety compliance, and safety participation. The agents with the greatest association with the probability of safety compliance were resilience, emotional exhaustion, and depersonalization and those with the greatest association with the probability of safety participation were personal accomplishment and resilience, respectively. Also, these findings revealed the strong role of resilience in mediating the associations between burnout factors and safety compliance.

### Practical applications

These results can be useful for policymakers so that risk assessments and measures for improving safety outcomes focus on each of the burnout dimensions and resilience. There is a wealth of evidence to show that timely interventions to improve working conditions and reduce burnout support both employees and the business, and that effective training programs can increase resilience skills in workers in the face of potentially dangerous events. Therefore, as detailed solutions and policies for improvement of safety behaviors, it is suggested:

- Employee resilience is strengthened through structured interventions such resilience training programs and peer support systems.
- Emotional exhaustion is mitigated to improve safety compliance through some measures such as job redesign for workload management and access to psychological services.
- Depersonalization is reduced to improve safety compliance through managerial and social interventions, such as leadership training in empathetic management and recognition and connection initiatives.
- Personal accomplishment is enhanced to foster safety participation through some plans, such as skill development programs, autonomy and role ownership, and feedback loops.
- Resilience and burnout metrics are incorporated into safety programs.

## Abbreviation:

EE: emotional exhaustion
DP: depersonalization
PA: personal accomplishment
SPSS: Statistical Package for the Social Sciences

## References

[1] Guo S, Tang B, Liang K, Zhou X, Li J. Comparative Analysis of the Patterns of Unsafe Behaviors in Accidents between Building Construction and Urban Railway Construction. J Constr Eng Manage. 2021;147(5). doi: 10.1061/(asce)co.1943-7862.0002013

[2] Seo D-C. An explicative model of unsafe work behavior. Safety Sci. 2005;43(3):187–211. doi: 10.1016/j.ssci.2005.05.001

[3] Neal A, Griffin MA, Hart PM. The impact of organizational climate on safety climate and individual behavior. Safety Sci. 2000;34(1–3):99–109. doi: 10.1016/s0925-7535(00)00008-4

[4] Spencer FC. Human error in hospitals and industrial accidents: current concepts. J Am Coll Surg. 2000;191(4):410–8. doi: 10.1016/s1072-7515(00)00691-8 11030247

[5] Wilkinson J, Lucas D. Better Alarm Handling — A Practical Application of Human Factors. Meas Control. 2002;35(2):52–4. doi: 10.1177/002029400203500204

[6] Hoff JW. The texaco incident. J Busin Ethics. 1987;6:365–9.

[7] Viner D. Occupational risk control: predicting and preventing the unwanted. Routledge. 2016.

[8] Janeway D. The Role of Psychiatry in Treating Burnout Among Nurses During the Covid-19 Pandemic. J Radiol Nurs. 2020;39(3):176–8. doi: 10.1016/j.jradnu.2020.06.004 32837392 PMC7377731

[9] Maslach C, Jackson SE. Burnout in organizational settings. Applied social psychology annual. 1984.

[10] Organization WH. Burn-out an “occupational phenomenon”: International Classification of Diseases. World Health Organization (WHO). 2019.

[11] Chen DW, Ren D. Behavior based safety (BBS) for accident prevention and positive study in construction enterprise. in 2015 International conference on management engineering and management innovation (ICMEMI-15). Atlantis Press; 2015.

[12] Mengenci C. Could burnout be a reason behind airlines accident? An empirical research study in Turkish Airlines companies. Hand. 2014;6(30).

[13] Van der Colff JJ, Rothmann S. Occupational stress, sense of coherence, coping, burnout and work engagement of registered nurses in South Africa. SA J Indust Psychol. 2009;35(1):1–10.

[14] Leiter MP, Gascón S, Martínez-Jarreta B. Making Sense of Work Life: A Structural Model of Burnout. J Applied Social Pyschol. 2010;40(1):57–75. doi: 10.1111/j.1559-1816.2009.00563.x

[15] Giusti EM, Pedroli E, D’Aniello GE, Stramba Badiale C, Pietrabissa G, Manna C, et al. The Psychological Impact of the COVID-19 Outbreak on Health Professionals: A Cross-Sectional Study. Front Psychol. 2020;11:1684. doi: 10.3389/fpsyg.2020.01684 32754102 PMC7366071

[16] Hoseinian H, Shirazi A. A study on the relationship between job stress and job burnout (at headquarters personnel of the post company). Appl Mathematics Eng Manag Technol. 2014;2(3):270–7.

[17] Xie Z, Wang A, Chen B. Nurse burnout and its association with occupational stress in a cross-sectional study in Shanghai. J Adv Nurs. 2011;67(7):1537–46. doi: 10.1111/j.1365-2648.2010.05576.x 21261698

[18] Liu H, Du Y, Zhou H. The Impact of Job Burnout on Employees’ Safety Behavior Against the COVID-19 Pandemic: The Mediating Role of Psychological Contract. Front Psychol. 2022;13:618877. doi: 10.3389/fpsyg.2022.618877 35282238 PMC8907840

[19] Bonanno GA. Clarifying and extending the construct of adult resilience. 2005.

[20] Chen Y, McCabe B, Hyatt D. Relationship between Individual Resilience, Interpersonal Conflicts at Work, and Safety Outcomes of Construction Workers. J Constr Eng Manage. 2017;143(8). doi: 10.1061/(asce)co.1943-7862.0001338

[21] Windle G. What is resilience? A review and concept analysis. Rev Clin Gerontol. 2010;21(2):152–69. doi: 10.1017/s0959259810000420

[22] Akbari M, et al. Development and validation of a resilience skills questionnaire for health sector professionals based on social cognitive theory. BioMed Res Int. 2024. 10.1155/2024/5660620 PMC10787653 38221911

[23] Youssef CM, Luthans F. Positive Organizational Behavior in the Workplace. J Manag. 2007;33(5):774–800. doi: 10.1177/0149206307305562

[24] Nahrgang JD, Morgeson FP, Hofmann DA. Safety at work: a meta-analytic investigation of the link between job demands, job resources, burnout, engagement, and safety outcomes. J Appl Psychol. 2011;96(1):71–94. doi: 10.1037/a0021484 21171732

[25] Vévoda J, et al. The relationship between psychological safety and burnout among nurses. Occupational Med. 2016;68.

[26] Shojafard J, et al. Relationship between burnout and resilience in emergency medicine personnel in Tehran. Quarterly Scient J Rescue Relief. 2014;6(2):0–0.

[27] Shakerinia I, Mohammadpour M. Relationship between job stress and resiliency with occupational burnout among nurses. J Kermanshah University Med Sci. 2010;14(2).

[28] Wehbe F, Al Hattab M, Hamzeh F. Exploring associations between resilience and construction safety performance in safety networks. Safety Sci. 2016;82:338–51.

[29] Li Y, Gao J, Qian C, Wu X. The Mediation Role of Safety Attitude in the Impact of Resilience on the Safety Behavior of Coal Miners in China. Int J Environ Res Public Health. 2022;19(22):15164. doi: 10.3390/ijerph192215164 36429880 PMC9690718

[30] Mittal A, Kassim A. Bayesian network technologies: applications and graphical models. IGI Global. 2007.

[31] Scanagatta M, Salmerón A, Stella F. A survey on Bayesian network structure learning from data. Prog Art Intell. 2019;8(4):425–39.

[32] Mohamadinejad A, Kakaei P, Nikdel T, Khalil Tahmasobi M, Tamoradi Mongenan N, Janizadeh R. Risk Identification and Risk Assessment Using Failure Mode and Effect Analysis in a Textile Industry. CJHR. 2019;4(3):60–5. doi: 10.29252/cjhr.4.3.60

[33] Nag PK, Nag A, Vyas H. Nonfatal accident analyses in the textile industry in India. Ergonomics Developing Regions. CRC Press. 2009. p. 281–90.

[34] Kaviani H, Khaghanizade M. The relationship between burnout and mental health among nurses. Tehran Univ Med J. 2007;65(6):65–75.

[35] Moalemi S, Kavosi Z, Beygi N, Deghan A, Karimi A, Parvizi MM. Evaluation of the Persian Version of Maslach Burnout Inventory-Human Services Survey among Iranian Nurses: Validity and Reliability. Galen Med J. 2018;7:e995. doi: 10.22086/gmj.v0i0.995 34466422 PMC8343696

[36] Connor KM, Davidson JRT. Development of a new resilience scale: the Connor-Davidson Resilience Scale (CD-RISC). Depress Anxiety. 2003;18(2):76–82. doi: 10.1002/da.10113 12964174

[37] Windle G, Bennett KM, Noyes J. A methodological review of resilience measurement scales. Health Qual Life Outcomes. 2011;9:8. doi: 10.1186/1477-7525-9-8 21294858 PMC3042897

[38] Derakhshanrad SA, et al. Standardization of connor-davidson resilience scale in iranian subjects with cerebrovascular accident. J Rehabilitation Sci Res. 2014;1(4):73–7.

[39] Mahdinia M, et al. Development and validation of a questionnaire for safety behavior assessment. 2016.

[40] Pilla RS. Alternative EM methods for nonparametric finite mixture models. Biometrika. 2001;88(2):535–50. doi: 10.1093/biomet/88.2.535

[41] Liu X, Huang G, Huang H, Wang S, Xiao Y, Chen W. Safety climate, safety behavior, and worker injuries in the Chinese manufacturing industry. Safety Sci. 2015;78:173–8. doi: 10.1016/j.ssci.2015.04.023

[42] Mohammadfam I, Ghasemi F, Kalatpour O, Moghimbeigi A. Constructing a Bayesian network model for improving safety behavior of employees at workplaces. Appl Ergon. 2017;58:35–47. doi: 10.1016/j.apergo.2016.05.006 27633196

[43] Cao Y, Fang X, Ottosson J, Näslund E, Stenberg E. A Comparative Study of Machine Learning Algorithms in Predicting Severe Complications after Bariatric Surgery. J Clin Med. 2019;8(5):668. doi: 10.3390/jcm8050668 31083643 PMC6571760

[44] Edú-Valsania S, Laguía A, Moriano JA. Burnout: A Review of Theory and Measurement. Int J Environ Res Public Health. 2022;19(3):1780. doi: 10.3390/ijerph19031780 35162802 PMC8834764

[45] Schaufeli WB, Buunk BP. Burnout: An overview of 25 years of research and theorizing. The handbook of work and health psychology. 2003. p. 282–424.

[46] Deldar K, Froutan R, Dalvand S, Gheshlagh RG, Mazloum SR. The Relationship between Resiliency and Burnout in Iranian Nurses: A Systematic Review and Meta-Analysis. Open Access Maced J Med Sci. 2018;6(11):2250–6. doi: 10.3889/oamjms.2018.428 30559897 PMC6290412

[47] Ríos-Risquez MI, García-Izquierdo M, Sabuco-Tebar E de LA, Carrillo-Garcia C, Martinez-Roche ME. An exploratory study of the relationship between resilience, academic burnout and psychological health in nursing students. Contemp Nurse. 2016;52(4):430–9. doi: 10.1080/10376178.2016.1213648 27436758

[48] Baier N, Roth K, Felgner S, Henschke C. Burnout and safety outcomes - a cross-sectional nationwide survey of EMS-workers in Germany. BMC Emerg Med. 2018;18(1):24. doi: 10.1186/s12873-018-0177-2 30126358 PMC6102842

[49] Salyers MP, Bonfils KA, Luther L, Firmin RL, White DA, Adams EL, et al. The Relationship Between Professional Burnout and Quality and Safety in Healthcare: A Meta-Analysis. J Gen Intern Med. 2017;32(4):475–82. doi: 10.1007/s11606-016-3886-9 27785668 PMC5377877

[50] Park S-Y, Eo Y-S. Mediating Effect of Ego-Resilience in the Relationship between Social Capital and Safety Awareness·Safety Pursuit Behavior of Adult Learner: Focusing on Changwon City. J Fisheries Marine Sci Educ. 2016;28(1):162–71. doi: 10.13000/jfmse.2016.28.1.162

[51] Corbeanu A, Iliescu D, Ion A, Spînu R. The link between burnout and job performance: a meta-analysis. European J Work Organizational Psychol. 2023;32(4):599–616. doi: 10.1080/1359432x.2023.2209320

[52] Zarei E, Khakzad N, Reniers G, Akbari R. On the relationship between safety climate and occupational burnout in healthcare organizations. Safety Sci. 2016;89:1–10. doi: 10.1016/j.ssci.2016.05.011

